# Diagnostic tool or screening programme? Asymptomatic testing for SARS-CoV-2 needs clear goals and protocols

**DOI:** 10.1016/j.lanepe.2020.100002

**Published:** 2020-11-13

**Authors:** Jordan P Skittrall, Mary D Fortune, Hamid Jalal, Hongyi Zhang, David A Enoch, Nicholas M Brown, Anne Swift

**Affiliations:** a Department of Applied Mathematics and Theoretical Physics, University of Cambridge, Centre for Mathematical Sciences, Wilberforce Road, Cambridge CB3 0WA, United Kingdom; b Department of Infectious Diseases, Cambridge University Hospitals NHS Foundation Trust, Addenbrooke's Hospital, Hills Road, Cambridge CB2 0QQ, United Kingdom; c Clinical Microbiology and Public Health Laboratory, Cambridge University Hospitals NHS Foundation Trust, Cambridge Biomedical Campus, Cambridge CB2 0QW, United Kingdom; d Department of Public Health and Primary Care, University of Cambridge, Institute of Public Health, Forvie Site, Cambridge Biomedical Campus, Cambridge CB2 0SR, United Kingdom; e Air Force Medical University, 169 Changle West Road, Xi'an, Shaanxi 710032, PR China

## 1 Introduction

Countries’ increases in testing capacity during the first waves of the COVID-19 pandemic, coupled with reductions in case numbers between waves, have resulted in shifts from diagnostic testing of symptomatic patients to mass screening. Viewed from the perspective of United Kingdom testing, it is therefore arguable that the largest asymptomatic testing programme, by testing rate, ever attempted in the country is currently being deployed. Other countries are deploying similar programmes. Even with the advent of further waves of infection, especially associated with northern hemisphere winter, asymptomatic testing is still occurring, and it can be anticipated that as waves recede and seasons change it will again increase. These shifts have led to policies that risk conflating diagnosis with screening.

Currently, there are local, regional and national variations in criteria for screening, in modes of delivery, in whether laboratories undertake confirmatory testing following positive screen results, in the extents of contact tracing undertaken, and in quality assurance of programmes, signifying discrepancies in the understanding and policy objectives of the screening being undertaken. Unavoidably, variations within the populations being tested, in terms of both disease prevalence (and hence the proportion of positive tests that are false positives) and the level of risk to the health of different individuals, also lead to implications for testing strategies, acceptability, and balancing the interests of individuals and society.

Screening for SARS-CoV-2 is being undertaken with heterogeneous inclusion criteria and with heterogeneous aims [1]. The UK government's early policy stated a primary aim of making diagnoses in symptomatic individuals, with the main aim of the testing strategy being to send back to work high risk critical workers in whom a diagnosis of COVID-19 was not made [2]. However, this was rapidly extended to testing people without symptoms in care homes or returning from hospital to care homes [3,4]. More recently, there are reports of government plans to increase UK SARS-CoV-2 testing capacity to 10 million tests per day – sufficient to test the entire population each week – with these plans mentioning both symptomatic individuals and their contacts [5]. Individual organisations have used increased testing capacity to test people without symptoms with the aim of reducing transmission of SARS-CoV-2 [6,7]. Asymptomatic screening has been adopted in hospital settings to guide both infection control practices around those with higher risk of being infectious and timings of treatment for other conditions (such as elective surgery and cancer chemotherapy) where there may be a higher risk of adverse outcomes if infected [8], [9], [10], [11]. It has been adopted in community settings to allow rapid isolation and cohorting of infectious individuals in facilities and hence to reduce morbidity and mortality from institutional outbreaks [12], [13], [14]. It has even been used to monitor the progress and guide timing of containment measures for an entire town [15]. Wider screening of healthcare workers [16] and university students and staff [17] has been advocated.

Testing strategies are being managed in a piecemeal fashion, but from a historical perspective this mirrors the introduction of many mass screening programmes. Heterogeneity within and between screening programmes for a single disease is not new [18,19], and as in historical cases when other screening was introduced in uncoordinated fashion, with the practice of screening ahead of evidence for its benefit, we now need to develop a systematic approach and ask to what ends we are screening, whether screening achieves these ends, and how we can approach screening methodically, in order that we can efficiently and economically achieve the best outcomes feasible as circumstances in the pandemic change. These are the purposes for which many countries have screening oversight organisations.

## 2 Not all positive tests reflect infection

**Fig. 1:**
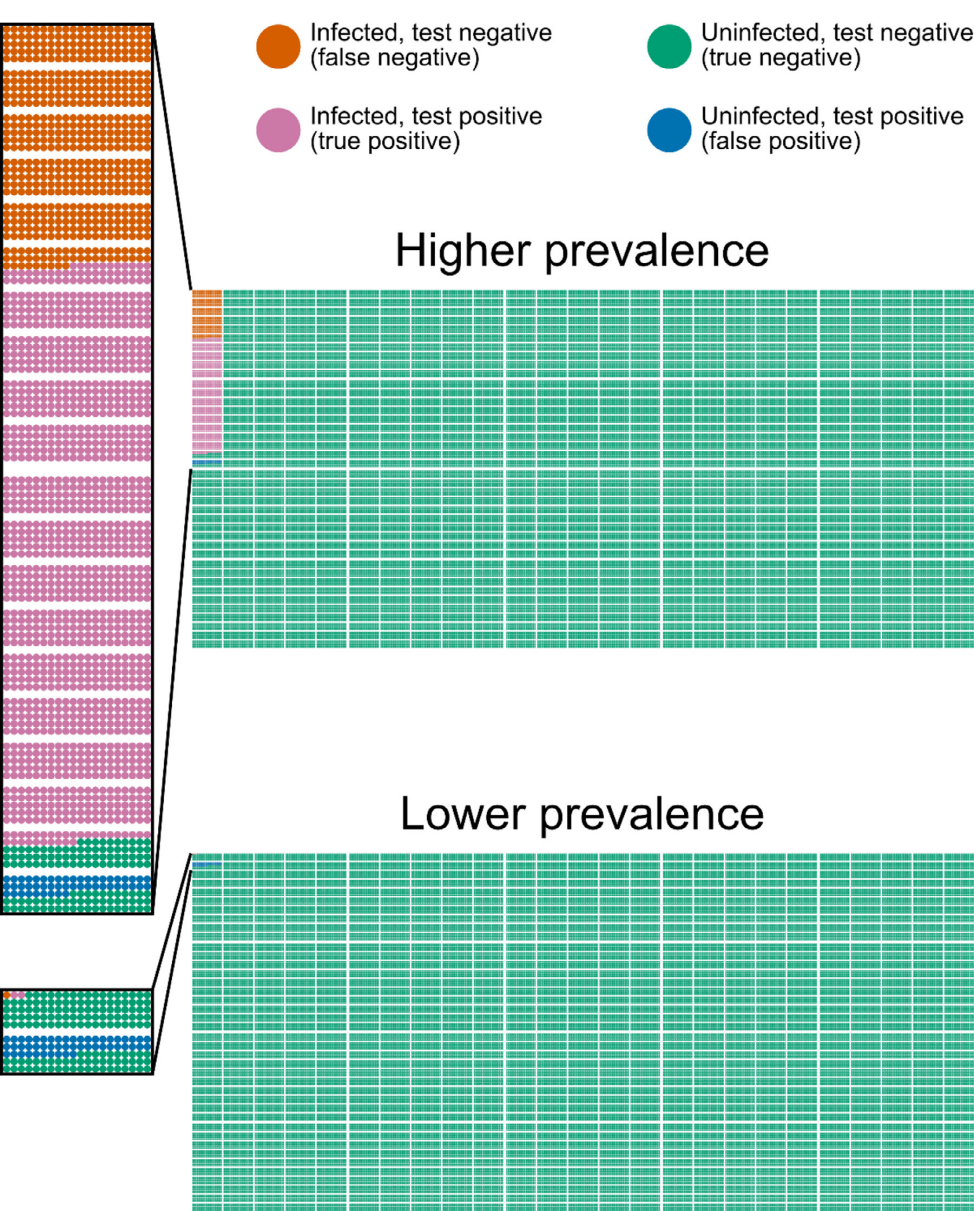
Pictogram representing outcomes of screening 100,000 people in (upper panel) higher prevalence setting (UK Office for National Statistics estimate of 1.33% test positivity in healthcare workers during period 27th April–10th May 2020 [30]) and (lower panel) lower prevalence setting (UK Office for National Statistics estimate of 0.052% test positivity in community during period 14th–27th June 2020 [31]), with sensitivity 70%, specificity 99.95%. To the left of each panel is an expanded view showing all true positive, false positive, and false negative results. Small rectangles represent 100 people, and medium rectangles represent 5000 people. In the top panel there are 1830 people with disease, of whom 1281 test positive (true positive) and 549 test negative (false negative), and 98,170 people without disease, of whom 49 test positive (false positive) and 98,121 test negative (true negative). The number needed to isolate to remove one infectious individual is 1.04. In the bottom panel there are 3 people with disease, of whom 2 test positive (true positive) and 1 tests negative (false negative), and 99,997 people without disease, of whom 50 test positive (false positive) and 99,947 test negative (true negative). The number needed to isolate to remove one infectious individual is 25.98. Note that using these data to generate full prevalence estimates with confidence intervals would require knowing the variability in sensitivity, which is difficult to determine.

Most tests for SARS-CoV-2 infection were developed and evaluated in the context of people with symptoms (i.e. to diagnose disease), whereas many of those now being tested are asymptomatic. Although a clinically significant proportion of those with infection have no symptoms (with estimates varying from under 50% to around 75%) [15,20,21], nevertheless the proportion of the entire asymptomatic general population with infection will be much smaller than the proportion of the symptomatic population with infection. Further, the absence of symptoms suggests different within-host viral dynamics and immune response, meaning it is not possible to extrapolate reliably from a test's performance in symptomatic individuals to ascertain a performance in asymptomatic individuals. It is difficult to evaluate a test's *sensitivity* (how well the test correctly identifies those with infection) in those without symptoms – especially in those who never develop symptoms – because there is no gold standard against which to compare. If (as suggested in the United Kingdom strategy) a test is used to move people from being isolated back into a situation where they may infect others, then sensitivity is important – and with an estimated test sensitivity around 70% [22], it appears *prima facie* that current test sensitivities may make this strategy risky; however, as sensitivity will correlate with degree of viral shedding and therefore infectivity, this theoretical risk is reduced in practice. (Sensitivity estimates in the literature are highly variable [23], [24], [25], measures are sometimes used to detect false negative results caused by inadequate sampling [26], and new tests with different performance characteristics are being introduced [27,28], meaning the interactions between test sensitivity and public health response need to be re-evaluated for each test whose deployment is considered.) Nevertheless, when a test is being used to identify and isolate asymptomatic infectious individuals who would otherwise have been free to infect others, any sensitivity that results in a clinically significant reduction in disease spread is useful (and a small percentage reduction in disease spread may be sufficient in populations where the virus effective reproduction number *R_eff_* is just above 1). In many ways, therefore, the test's *specificity* (how well the test correctly excludes those without infection) matters more in largely-asymptomatic populations: when the prevalence of infection is low, even a highly specific test results in many of the positive results – perhaps even the majority – coming from those without infection (false positives), reflecting the preponderance of individuals in that population without infection. Just as with sensitivity, the lack of gold standard makes quantifying the specificity of a SARS-CoV-2 diagnostic test difficult, but we have shown that when the prevalence of infection is low it is possible to make reliable estimates [29]. The issue of positive tests in those without infection becomes prominent for *any* test when population prevalence is sufficiently low, but with realistic estimates of a test sensitivity of 70% [22] (note that most of the loss of sensitivity comes at time of sampling, not during laboratory testing) and a test specificity of 99.95% [29], it is probable that during the summer of 2020, the United Kingdom reached a point where reported SARS-CoV-2 positivity rates mostly represented false positive tests, with week-to-week variations largely representing natural fluctuations in false positive rates (Fig. 1).

## 3 What do we do when people have positive tests?

In a screening programme, usually the next step after the initial screening test is to conduct confirmatory testing for those with positive tests. Nucleic acid amplification testing is highly specific, but even this high specificity is insufficient when used at high throughput in groups with a low prevalence of infection (Fig. 1). Steps that virologists usually deploy to improve single test specificity, such as expert review of results, do not apply to some of the new amplification technologies in use, do not scale to the number of tests currently being undertaken, and still do not help in some difficult cases and some uncommon modes of error occasionally seen at high throughput (such as transposing labels on samples or contamination of a sample with positive control material). As a result, confirmatory testing is advisable, and we have therefore begun to deploy confirmatory testing in England [32]. With SARS-CoV-2 testing, such confirmation is most likely to involve repeating the original test, or running it on a different testing platform (an approach similar to that taken in existing screening programmes such as antenatal HIV serological testing).

In the case of SARS-CoV-2 infection, the short time during which infection is asymptomatic but infectious to others (whether presymptomatic, or in those who never develop symptoms) means there is a trade-off between accuracy and timeliness not seen in other screening programmes. Undertaking confirmatory testing of positive screens decreases the chance of an overall positive test in a person without infection, but the additional turnaround time required for confirmatory testing covers the period in which the highest benefit from isolating the person may be obtained [33]. The obvious answer to this issue is to request that those positive on initial screens isolate whilst confirmatory testing is undertaken. Implementing such a protocol requires a high level of understanding of the risks being managed amongst those tested and those advising on actions to be taken: experience has shown that full compliance with self-isolation instructions already occurs in only a minority [34]. The increased use of confirmatory testing for SARS-CoV-2 will be accompanied by an urgent need for professional education of non-specialists, particularly focussed on safeguarding those awaiting confirmatory testing from inadvertent infection, on clearly explaining processes so as to maintain public trust, and on having the courage to de-escalate those whose positive screening tests are not confirmed.

This leads us to fundamental issues that go beyond the analytical performance of a test and the factors affecting that analytical performance. When a test is used for screening, as opposed to diagnosis, several other considerations come into effect, apart from the simple one of whether the screening test has managed to reach a correct diagnosis. Chiefly, we need strategies for managing people with positive test results – including those with positive screening tests awaiting confirmation. Such strategies need to maximise benefits from reducing infectiousness, whilst minimising the varied harms that can result from a positive test. Screening will only yield benefit if, in addition to infectious individuals, some non-infectious individuals are asked to isolate, and careful analysis and good communication of such strategy options and their alternatives is ethically and practically necessary to ensure a net benefit from screening and retain public confidence in the pandemic response.

## 4 Benefits, harms, and dilemmas

In the community (including the setting of healthcare staff screening), there is a need to consider whether it is acceptable to have a situation where people will be required to isolate, either whilst awaiting confirmatory testing, or (if there is insufficient capacity for confirmatory testing) for the entire duration of potential infectiousness, with the possibility of no benefit to anybody because they are not in fact infected. Such isolation may entail inconvenience, psychological distress, disruption of family life, loss of earnings, and wider disruption to economic activity. It is especially important for medical professionals, in a relatively secure socioeconomic position, not to overlook the reality that some people being asked to isolate need to balance their perception of the risk from COVID-19 with a risk that isolation will involve a loss of job, and consequently possibly even housing and the ability to afford to feed themselves and their families. Disengagement from screening or follow-up is not a problem unique to SARS-CoV-2 screening, and in addressing it there are lessons to be learned from studies of those who do not attend screening or follow-up in other programmes, such as normalising discussions about screening within social groups, addressing individual fatalism about infection, addressing negative perceptions of the particular programme, sending reminders, simplifying the testing experience, providing help to mitigate the short-term drawbacks of screening and emphasising the long-term benefits [35], [36], [37], [38]. In the hospital or care setting, an individual with a positive screen but not infected may be placed at greater risk of nosocomial infection if cohorted with others with or at higher risk of infection, or may miss out on essential procedures that are delayed because of potential infectiousness. In the care setting, isolation or cohorting may involve removing a person from his or her usual place of residence.

The best way to manage positive tests may also depend upon the prevalence of disease. Regardless of disease prevalence, most of the potential harm to an individual (from isolation and its consequences or from delayed access to other healthcare) remains the same. However, the expected benefit changes depending upon disease prevalence: when there are many people with disease, then the chances of somebody with a positive screen having infection are higher, so the expected benefit to the individual and to others if the person is managed as infected is higher, so this management is more justifiable. Conversely, when there are few people with disease, and so a person with a positive screen has a relatively low chance of being infected, the expected benefit from managing the individual as infected is lower, the benefit may be outweighed by the risks, and it is harder to justify risking harm to that individual in order to protect others. The major exception to this paradigm is where a region is pursuing an elimination strategy, and so the expected benefits from avoiding single infections in a low prevalence setting are higher, and hence may still outweigh the risks of managing individuals as infected. As a result, it is important for those making public health and infection control decisions to make the distinction, ethically and practically, between measures intended to keep disease prevalence low and measures intended to eliminate disease altogether.

In all these settings there is a practical and ethical dilemma in that everybody's overall risk of harm is reduced if enough people are willing to be tested and isolated if the test is positive, but each person who undergoes testing incurs a small risk of harm to themselves from a positive test. People's responses to screening invitations will depend upon their understanding of and attitude to the test itself [35], [36], [37], [38]. Their responses may be impacted by others’ uptake of testing and behaviour relating to possible infection, because the behaviour of others may impact upon perceived societal obligation [39]. Their willingness to isolate may be affected by social and economic factors [40,41], and the possibility of modifying these factors (such as undertaken in the United Kingdom by introducing a payment to some on low incomes who are asked to isolate [42]) and the details of how raise behavioural, economic, political, and ethical questions that cannot be detached and considered separately from the properties of tests being undertaken. Different individuals will accept different thresholds of risk (e.g. a healthcare worker facing being sent home on full pay from a ward full of vulnerable patients, a university student positive on a pooled screen facing isolation in a bedsit with a shared bathroom, and a self-employed worker with no clear potential exposure facing complete loss of income but still needing to pay rent are in very different situations), so it is challenging to determine the optimal overall strategy for reducing infection. Selecting and employing appropriate risk communication strategies, learned from previous work in screening, will be a key component [43].

Those recommending how to act on results must recognise that people being asked to take measures harmful to themselves during a low prevalence phase may eventually exhibit a response similar to that of the villagers in the story of The Boy Who Cried Wolf, where the repeated raising of false alarms eventually means there is no response to a real emergency [44]. If some of those required to isolate feel well and perceive they pose low risk to others, this may reduce the likelihood that they will follow public health measures and so reduce the effectiveness of those measures in stopping infection spreading. Such perceptions may persist into a pandemic phase where the prevalence of infection is higher, reducing the later effectiveness of responses to waves of infection. The key here is good communication from those leading the public health response. Honest communication with people that they are being asked to isolate even though they might be uninfected not only respects their autonomy – an issue that has arisen in the past with communication within screening programmes [45] – but also lays the groundwork for the possibility that they, or people close to them, might later be asked to isolate again.

A further issue is that the addition of uninfected individuals to surveillance data may make it harder to trace contacts of infected individuals, and generates statistical noise (additional variability in recorded infection rates), making it harder to detect and respond to increases in infection rates. Even if results arrive too late to impact other management decisions, confirmatory testing may still be valuable, since it may enable us to reduce this noise. To reduce statistical noise, repeat sampling for nucleic acid amplification testing, or serological testing, may help, but nucleic acid amplification testing on a repeat sample may suffer from insensitivity at time of sampling (an issue that has already been overcome if testing is repeated on the initial sample), and serological testing for recent infection is a separate heterogeneous field with its own issues of sensitivity and specificity, in addition to timing of seroconversion meaning these tests have limited utility for identifying infectious individuals [10,46]. For these reasons, repeat nucleic acid amplification testing on the original sample is most likely to minimise statistical noise.

## 5 We already have frameworks for thinking about screening programmes

The development of strategies for managing positive screen results is the major issue as we move from diagnostic testing for SARS-CoV-2 to screening, but there are further factors to consider. Many authors have produced criteria to describe appropriate screening programmes, perhaps most famously Wilson and Jungner for the World Health Organization [47]; an example of a modern set of criteria is the set used by the United Kingdom National Screening Committee (Panel 1). Evaluation of screening for SARS-CoV-2 in asymptomatic individuals should be considered in light of each of these, or similar, criteria, and in light of our experiences of screening programme governance [19,49]. It is particularly instructive to consider how, with regard to these criteria, screening for SARS-CoV-2 differs from screening for other conditions, and what the implications for a screening programme are in light of these differences.

**Panel 1:** 

**The condition** 1. The condition should be an important health problem as judged by its frequency and/or severity. The epidemiology, incidence, prevalence, and natural history of the condition should be understood, including development from latent to declared disease and/or there should be robust evidence about the association between the risk or disease marker and serious or treatable disease. 2. All the cost-effective primary prevention interventions should have been implemented as far as possible. 3. If the carriers of a mutation are identified as a result of screening the natural history of people with this status should be understood, including the psychological implications. **The test** 4. There should be a simple, safe, precise, and validated screening test. 5. The distribution of test values in the target population should be known and a suitable cut-off level defined and agreed. 6. The test, from sample collection to delivery of results, should be acceptable to the target population. 7. There should be an agreed policy on the further diagnostic investigation of individuals with a positive test result and on the choices available to those individuals. 8. If the test is for a particular mutation or set of genetic variants the method for their selection and the means through which these will be kept under review in the programme should be clearly set out. **The intervention** 9. There should be an effective intervention for patients identified through screening, with evidence that intervention at a presymptomatic phase leads to better outcomes for the screened individual compared with usual care. Evidence relating to wider benefits of screening, for example those relating to family members, should be taken into account where available. However, where there is no prospect of benefit for the individual screened then the screening programme should not be further considered. 10. There should be agreed evidence based policies covering which individuals should be offered interventions and the appropriate intervention to be offered. **The screening programme** 11. There should be evidence from high quality randomised controlled trials that the screening programme is effective in reducing mortality or morbidity. Where screening is aimed solely at providing information to allow the person being screened to make an “informed choice” (such as Down's syndrome or cystic fibrosis carrier screening), there must be evidence from high quality trials that the test accurately measures risk. The information that is provided about the test and its outcome must be of value and readily understood by the individual being screened. 12. There should be evidence that the complete screening programme (test, diagnostic procedures, treatment/intervention) is clinically, socially and ethically acceptable to health professionals and public. 13. The benefit gained by individuals from the screening programme should outweigh any harms, for example from overdiagnosis, overtreatment, false positives, false reassurance, uncertain findings and complications. 14. The opportunity cost of the screening programme (including testing, diagnosis and treatment, administration, training and quality assurance) should be economically balanced in relation to expenditure on medical care as a whole (value for money). Assessment against this criterion should have regard to evidence from cost benefit and/or cost effectiveness analyses and have regard to the effective use of available resource. **Implementation criteria** 15. Clinical management of the condition and patient outcomes should be optimised in all health care providers prior to participation in a screening programme. 16. All other options for managing the condition should have been considered (such as improving treatment or providing other services), to ensure that no more cost effective intervention could be introduced or current interventions increased within the resources available. 17. There should be a plan for managing and monitoring the screening programme and an agreed set of quality assurance standards. 18. Adequate staffing and facilities for testing, diagnosis, treatment and programme management should be available prior to the commencement of the screening programme. 19. Evidence-based information, explaining the purpose and potential consequences of screening, investigation and preventative intervention or treatment, should be made available to potential participants to assist them in making an informed choice. 20. Public pressure for widening the eligibility criteria, for reducing the screening interval, and for increasing the sensitivity of the testing process, should be anticipated. Decisions about these parameters should be scientifically justifiable to the public.

United Kingdom National Screening Committee criteria for appraising the viability, effectiveness and appropriateness of a screening programme[48] (Crown copyright; contains public sector information licensed under the Open Government Licence v3.0).

There are 11 population screening programmes currently approved in the United Kingdom [50]. Of these, only two relate to conditions caused by infectious diseases (cervical cancer and infectious diseases in pregnancy). In all current programmes, the population at risk is smaller than for SARS-CoV-2 and the pre-symptomatic stage of the disease (criterion 9) lasts for much longer than the few days in which maximum benefit can be derived from isolating those with asymptomatic SARS-CoV-2 infection. This means that in current programmes much longer screening intervals and times for decisions on results can be allowed than work for SARS-CoV-2. All current screening programmes, including those for infectious diseases, are intended for the direct benefit of those screened or their offspring (criterion 9), whereas the main benefit of screening for SARS-CoV-2 is to others in a population. All current treatments for SARS-CoV-2 infection are supportive or validated in those with symptoms (i.e. with COVID-19; criteria 9–11) [51], [52], [53], and when a person has symptoms there is usually time to test for SARS-CoV-2 without substantially affecting management, so there is little to no benefit to the person screened in being tested prior to symptom development, and indeed inconvenience and possibly even harm (criterion 13). The importance of COVID-19 as a health problem (criterion 1), given its pandemic status, is likely to justify the cost of case finding (criteria 2, 14, 16), especially given that case finding has the potential to prevent further cases. Overall, SARS-CoV-2 screening needs to be larger in scale than other programmes, with faster turnaround from testing to decisions on management and adequate resolution of discussions on the potential consequences to individuals and populations from being screened.

## Conclusions

In short, because the best approach to screening depends on situation – and particularly upon population prevalence of infection, the sensitivity and specificity of different tests, ability to get results back quickly enough to make a difference, and different people's willingness to accept personal inconvenience and harm – there is likely to be no one-size-fits-all best solution to the question of how to screen. But this heterogeneity of circumstance should lead to even more care in optimising screening wherever it is used.

As countries’ capacities for SARS-CoV-2 testing increase, and screening of asymptomatic individuals becomes feasible, it is highly desirable to repurpose existing screening oversight organisations, to leverage their longstanding experience in targeting screening tests to gain maximum benefit from available capacity. When the number of cases of COVID-19 declines in a community, it is crucial to update triage guidelines, written for a different pandemic phase, with a different understanding of the natural history of infection and infectivity, and different testing availability, aimed at determining the infection status of symptomatic individuals. Such guideline updates must specify triggers for further revision should the community prevalence of SARS-CoV-2 infection either surge or decline further. As screening programmes become more established, procedures to evaluate their effectiveness in target populations should be implemented, and studies to determine how to increase effectiveness undertaken. As well as the technical aspects and biomedical consequences of testing, these studies should aim to understand population engagement with screening, and social and economic impacts.

The history of screening is already replete with examples where at best more good could have been done, and at worst unnecessary harm was caused, because an overenthusiastic belief that more testing is always better led to unwillingness to allow critical appraisal of programmes. In the middle of a pandemic, we must learn this lesson from history rapidly.

## Contributors

Jordan P. Skittrall: conceptualisation, data analysis, figures, writing – original draft; Mary D. Fortune: conceptualisation, writing – original draft; Hamid Jalal: conceptualisation; Hongyi Zhang: conceptualisation; David A. Enoch: conceptualisation; Nicholas M. Brown: conceptualisation; Anne Swift: conceptualisation, writing – review and editing.

## Declaration of Competing Interest

Jordan P. Skittrall is funded by the Mason Medical Research Foundation. The authors declare no other conflicts of interests.

## References

[1] Multidisciplinary task and finish group on mass testing for the Scientific Advisory Group on Emergencies (27 August 2020). https://www.gov.uk/government/publications/tfms-consensus-statement-on-mass-testing-27-august-2020.

[2] Department of Health and Social Care (2020). https://www.gov.uk/government/publications/coronavirus-covid-19-scaling-up-testing-programmes.

[3] Department of Health and Social Care (2020). https://www.gov.uk/government/publications/coronavirus-covid-19-adult-social-care-action-plan.

[4] Hancock M., Whatley H, Department of Health and Social Care (2020). https://www.gov.uk/government/news/regular-retesting-rolled-out-for-care-home-staff-and-residents.

[5] Iacobucci G., Coombes R (2020). Covid-19 : government plans to spend £100bn on expanding testing to 10 million a day. BMJ.

[6] Rivett L., Sridhar S., Sparkes D. (2020). Screening of healthcare workers for SARS-CoV-2 highlights the role of asymptomatic carriage in COVID-19 transmission. Elife.

[7] Eyre D.W., Lumley S.F., Donnell D.O. (2020). Differential occupational risks to healthcare workers from SARS-CoV- 2 observed during a prospective observational study. Elife.

[8] Al-Shamsi H.O., Coomes E.A., Alrawi S (2020). Screening for COVID-19 in asymptomatic patients with cancer in a hospital in the United Arab Emirates. JAMA Oncol.

[9] Sutton D., Fuchs K., D'Alton M., Goffman D (2020). Universal screening for SARS-CoV-2 in women admitted for delivery. N Engl J Med.

[10] Lahner E., Dilaghi E., Prestigiacomo C. (2020). Prevalence of Sars-Cov-2 infection in health workers (HWs) and diagnostic test performance: the experience of a teaching hospital in central Italy. Int J Environ Res Public Health.

[11] Okonkwo I.N.C., Howie A., Parry C. (2020). The safety of paediatric surgery between COVID-19 surges: an observational study. Anaesthesia.

[12] Dora A V., Winnett A., Jatt L.P. (2020). Universal and Serial Laboratory Testing for SARS-CoV-2 at a Long-Term Care Skilled Nursing Facility for Veterans — Los Angeles, California, 2020. MMWR Morb Mortal Wkly Rep.

[13] Njuguna H., Wallace M., Simonson S. (2020). Serial Laboratory Testing for SARS-CoV-2 Infection Among Incarcerated and Detained Persons in a Correctional and Detention Facility - Louisiana, April-May 2020. MMWR Morb Mortal Wkly Rep.

[14] Danis K., Fonteneau L., ECDC Public Health Emergency Team (2020). High impact of COVID-19 in long-term care facilities, suggestion for monitoring in the EU/EEA, May 2020. Eurosurveillance.

[15] Lavezzo E., Franchin E., Ciavarella C. (2020). Suppression of a SARS-CoV-2 outbreak in the Italian municipality of Vo’. Nature.

[16] Black J.R.M., Bailey C., Przewrocka J., Dijkstra K.K., Swanton C (2020). COVID-19: the case for health-care worker screening to prevent hospital transmission. Lancet.

[17] Independent SAGE (2020). https://www.independentsage.org/wp-content/uploads/2020/09/Universities-late-Sept-statement-28-9-20-final.pdf.

[18] Williams J.H., Carter S.M., Rychetnik L (2014). ‘Organised’ cervical screening 45 years on: how consistent are organised screening practices?. Eur J Cancer.

[19] Raffle A.E., Mackie A., Gray J.A.M (2019).

[20] Emery J.C., Russell T.W., Liu Y. (2020). The contribution of asymptomatic SARS-CoV-2 infections to transmission on the Diamond Princess cruise ship. Elife.

[21] Ladhani S.N., Jeffery-Smith A.J., Patel M. (2020). High prevalence of SARS-CoV-2 antibodies in care homes affected by COVID-19 a prospective cohort study in England. medRxiv.

[22] Woloshin S., Patel N., Kesselheim A.S (2020). False negative tests for SARS-CoV-2 infection — Challenges and implications. N Engl J Med.

[23] Yang Y., Yang M., Shen C. (2020). Evaluating the accuracy of different respiratory specimens in the laboratory diagnosis and monitoring the viral shedding of 2019-nCoV infections. medRxiv.

[24] Arevalo-Rodriguez I., Buitrago-Garcia D., Simancas-Racines D. (2020). False-negative results of initial RT-PCR assays for COVID-19: a systematic review. medRxiv.

[25] Zhao J., Yuan Q., Wang H. (2020). Antibody responses to SARS-CoV-2 in patients of novel coronavirus disease 2019. Clin Infect Dis.

[26] Yan Y., Chang L., Wang L (2020). Laboratory testing of SARS-CoV, MERS-CoV, and SARS-CoV-2 (2019-nCoV): current status, challenges, and countermeasures. Rev Med Virol.

[27] Rödel J., Egerer R., Suleyman A. (2020). Use of the variplex™ SARS-CoV-2 RT-LAMP as a rapid molecular assay to complement RT-PCR for COVID-19 diagnosis. J Clin Virol.

[28] Collier D., Assennato S., Sithole N. (2020). Rapid point of care nucleic acid testing for SARS-CoV-2 in hospitalised patients: a clinical trial and implementation study. Cell Reports Med.

[29] Skittrall J.P., Wilson M., Smielewska A.A. (2020). Specificity and positive predictive value of SARS-CoV-2 nucleic acid amplification testing in a low prevalence setting. Clin Microbiol Infect.

[30] Office for National Statistics. Coronavirus (COVID-19) infection survey pilot: England, 14 May 2020. https://www.ons.gov.uk/peoplepopulationandcommunity/healthandsocialcare/conditionsanddiseases/bulletins/coronaviruscovid19infectionsurveypilot/england14may2020.

[31] Office for National Statistics. Coronavirus (COVID-19) infection survey pilot: 2 July 2020. https://www.ons.gov.uk/peoplepopulationandcommunity/healthandsocialcare/conditionsanddiseases/bulletins/coronaviruscovid19infectionsurveypilot/2july2020.

[32] Public Health England. Assurance of SARS-CoV-2 rna positive results during periods of low prevalence. https://www.gov.uk/government/publications/sars-cov-2-rna-testing-assurance-of-positive-results-during-periods-of-low-prevalence/assurance-of-sars-cov-2-rna-positive-results-during-periods-of-low-prevalence.

[33] He X., Lau E.H.Y., Wu P. (2020). Temporal dynamics in viral shedding and transmissibility of COVID-19. Nat Med.

[34] Smith L.E., Potts H.W.W., Amlȏt R., Fear N.T., Michie S., Rubin J (2020). Adherence to the test, trace and isolate system: results from a time series of 21 nationally representative surveys in the UK (the COVID-19 Rapid Survey of Adherence to Interventions and Responses [CORSAIR] study). medRxiv.

[35] Palmer C.K., Thomas M.C., Von Wagner C., Raine R (2014). Reasons for non-uptake and subsequent participation in the NHS Bowel Cancer Screening Programme: a qualitative study. Br J Cancer.

[36] Crockett R., Wilkinson T.M., Marteau T.M (2008). Social patterning of screening uptake and the impact of facilitating informed choices: psychological and ethical analyses. Heal Care Anal.

[37] Valent F., Sammartano F., Degano S. (2020). Reasons for non-participation in public oncological screening programs in the Italian region Friuli Venezia Giulia. Public Health.

[38] Sarma E.A., Silver M.I., Kobrin S.C., Marcus P.M., Ferrer R.A (2019). Cancer screening: health impact, prevalence, correlates, and interventions. Psychol Heal.

[39] Mahase E (2020). Covid-19: UK government's defence of senior aide has damaged public and NHS confidence, say experts. BMJ.

[40] Bauerle Bass S., Ruzek S.B., Ward L. (2010). If you ask them, will they come? Predictors of quarantine compliance during a hypothetical avian influenza pandemic: results from a statewide survey. Disaster Med Public Health Prep.

[41] Bodas M., Peleg K. (2020). Self-isolation compliance in the COVID-19 era influenced by compensation: findings from a recent survey in Israel. Health Aff.

[42] Department of Health and Social Care (2020). https://www.gov.uk/government/publications/test-and-trace-support-payment-scheme-claiming-financial-support/claiming-financial-support-under-the-test-and-trace-support-payment-scheme.

[43] Biesecker B.B., Schwartz M.D., Marteau T.M (2013). Enhancing informed choice to undergo health screening: a systematic review. Am J Health Behav.

[44] Aesop's Fables.

[45] Thornton H (1999). Evidence-based information. Lancet.

[46] Deeks J.J., Dinnes J., Takwoingi Y. (2020). Antibody tests for identification of current and past infection with SARS-CoV-2. Cochrane Database Syst Rev.

[47] Wilson J., Jungner G. Principles and practice of screening for disease. WHO Public Paper 34. 1968.

[48] Public Health England. Criteria for appraising the viability, effectiveness and appropriateness of a screening programme. https://www.gov.uk/government/publications/evidence-review-criteria-national-screening-programmes/criteria-for-appraising-the-viability-effectiveness-and-appropriateness-of-a-screening-programme.

[49] Sturdy S., Miller F., Hogarth S. (2020). Half a Century of Wilson & Jungner: reflections on the Governance of Population Screening. Wellcome Open Res.

[50] Population screening programmes. https://www.gov.uk/topic/population-screening-programmes.

[51] The RECOVERY Collaborative Group (2020). Dexamethasone in Hospitalized Patients with Covid-19 — Preliminary Report. N Engl J Med.

[52] Wang Y., Zhang D., Du G. (2020). Remdesivir in adults with severe COVID-19: a randomised, double-blind, placebo-controlled, multicentre trial. Lancet.

[53] Beigel J.H., Tomashek K.M., Dodd L.E. (2020). Remdesivir for the treatment of Covid-19 — Preliminary report. N Engl J Med.

